# The Mechanism of Mori Folium and Eucommiae Cortex against Cyclophosphamide-Induced Immunosuppression Integrating Network Pharmacology, Molecular Docking, Molecular Dynamics Simulations, and Experimental Validation

**DOI:** 10.3390/metabo13111151

**Published:** 2023-11-15

**Authors:** Jinde Liu, Qiao Rong, Chunxiao Zhang, Ali Tariq, Lin Li, Yongning Wu, Feifei Sun

**Affiliations:** 1 Animal-Derived Food Safety Innovation Team, Anhui Agricultural University, Hefei 230036, China; ljd971225@stu.ahau.edu.cn (J.L.); rongq@stu.ahau.edu.cn (Q.R.); zhang_cx0412@stu.ahau.edu.cn (C.Z.); lilin@ahau.edu.cn (L.L.); 2 College of Veterinary Sciences, University of Agriculture Peshawar, Peshawar 17131, Pakistan; tariq.ali.khattak@aup.edu.pk; 3 NHC Key Laboratory of Food Safety Risk Assessment, China National Center for Food Safety Risk Assessment, Beijing 100017, China

**Keywords:** Mori Folium, Eucommiae Cortex, immunosuppression, network pharmacology, molecular docking, molecular dynamics simulations

## Abstract

It has been reported that Mori Folium (MF) and Eucommiae Cortex (EC) exhibit pharmacological effects in the treatment of immunosuppression. However, the mechanism of MF and EC against immunosuppression remains unclear. This study aims to explore the mechanism of action of MF and EC for the treatment of immunosuppression through network pharmacology, molecular docking, molecular dynamics simulations and animal experiments. As a result, 11 critical components, 9 hub targets, and related signaling pathways in the treatment of immunosuppression were obtained based on network pharmacology. The molecular docking suggested that 11 critical components exhibited great binding affinity to 9 hub targets of immunosuppression. The molecular dynamics simulations results showed that (-)-tabernemontanine-AR, beta-sitosterol-AR and Dehydrodieugenol-HSP90AA1 complexes are stably bound. Additionally, in the animal experiments, the treated group results compared to the control group suggest that MF and EC have a significant effect on the treatment of immunosuppression. Therefore, MF and EC treatment for immunosuppression may take effects in a multi-component, multi-target, and multi-pathway manner. The results herein may provide novel insights into the treatment of immunosuppression in humans.

## 1. Introduction

The immune system plays an important role in countering infections and tumors; it acts as the body’s defense system by protecting our body cells, tissues, and organs from invading infections from harmful microorganisms and other disease-causing microbes [1,2]. Immunomodulation is the interaction between immune cells and immune molecules in the immune system, as well as with other systems such as the neuroendocrine system, used to maintain the body at the most appropriate level. It involves two major mechanisms: immune-stimulation and immunosuppression [3], where the immunosuppression refers to the inhibition of the immune response and is becoming increasingly common, especially when patients have received transplanted organs or bone marrow [2].

*Morus alba* L., belonging to the Moraceae family, is one of the most valuable plants, and rich in natural products; its pharmaceutical name is Mori Folium (MF) [4,5]. Mori Folium contains a variety of active ingredients, such as polysaccharides [6], flavonoids [7], alkaloids [8], etc. The comprehensive effects of these active ingredients reflect the pharmacological effects of MF, including antidiabetic, anti-inflammatory, antibacterial, cardiovascular and cardioprotective, hypolipidemic, antioxidant, and antiatherogenic abilities [9,10].

Eucommiae Cortex (EC) is derived from the dried bark of Eucommia ulmoides Oilv, wherein active compounds are separated and analyzed using modern chemical methods; a total of 112 compounds have been identified, mainly including lignans, iridoids, phenolics, steroids, terpenoids, and flavonoids [11]. Currently, many studies have shown that the pharmacological effects of EC are antihypertensive, hypolipidemic, anti-obesity, antidiabetic, neuroprotective, antioxidative, antifatigue, anti-aging, antitumor, anti-inflammatory and immune-function-enhancing [11].

Network pharmacology is a new field combining traditional Chinese medicine (TCM) and network pharmacology, which is based on the theory of system biology and the viewpoint of network pharmacology. Network pharmacology facilitates under-standing of the compatibility law of prescriptions, identification of TCM medicine, prediction of disease-related targets, and the action mechanism of TCM [12]. Molecular docking can be used to reveal biomolecular interactions and mechanisms. Currently, a variety of docking methods are available for computational docking, wherein limitations such as simplified scoring function are obvious. The related reference demonstrated that virtual screening might also lead to inactive molecules, which were randomly chosen. However, it also concluded that molecular dynamics simulations may be an alternative method, counteracting some limitations and inactive molecules [13]. In the present study, the natural products selected for study, including the active ingredients present in the natural products, have been more or less demonstrated in preliminary experiments to have important pharmacological effects on the immunomodulation of the organism. In addition, these innovative molecular methods used to explore Mori Folium and Eucommiae Cortex against cyclophosphamide-induced immunosuppression are not random, but are further adopted on the basis of animal experiments demonstrating resistance to immunosuppression. In this study, network pharmacology, molecular docking, and molecular dynamics simulation methods were used to explore the pharmacological and molecular mechanisms of the anti-immunosuppression activity of Mori Folium and Eucommiae Cortex (MFEC) extracts. Additionally, the underlying mechanism of MFEC may provide a new insight into the screening of potential bioactivity, and may facilitate the development of drugs for anti-immunosuppression treatment from the active compounds.

## 2. Materials and Methods

### 2.1. Animal Experiments

The animals were housed in the animal house of Anhui Agricultural University, with commercial standard diets and water ad libitum. Additionally, among the experimented animals, half are males and the other half are females. In the current study, all animal experiments completely comply with the ARRIVE guidelines and were conducted in accordance with the U.K. Animals (Scientific Procedures) Act, 1986 and the associated guidelines, the EU Directive 2010/63/EU for animal experiments, and the National Research Council’s Guide for the Care and Use of Laboratory Animals.

In this study, fifty mice were randomly divided into five groups, with each group containing ten mice: control group, model group, MF group, EC group and MFEC group. After 7 d accommodation, on the 8th–10th day, all groups except the control group were intraperitoneally injected with cyclophosphamide at a rate of 80 mg/kg once a day. The control group was injected with the same amount of normal saline. On the 11th–17th day, the MF group, EC group and MFEC group were fed 200 mg/kg MF, EC, MFEC, respectively. The other groups were fed the same amount of normal saline. All the experiments pertaining to animals comply with the commonly accepted “3R”, according to the guidelines of Anhui Agricultural University regarding the protection of animals. The spleens of the mice were collected and weighted (g), and the spleen index of the mice was calculated as follows:

Spleen index (%) = spleen weight (g)/body mass of mice (g) × 100%

Additionally, the IF-2, IF-6 and TNF-α levels in serum were determined according to ELISA kits for mice (Shanghai Jianglai Biotechnology Co., Ltd., Shanghai, China), and the 100 µL of negative control, standard, or diluted serum sample to be tested were added to the microtiter plate to make duplicate wells. The microtiter plate was covered with film, placed horizontally, and incubated at room temperature for 60 min. After discarding the liquid, the plate was washed four times with washing solution an dried after each operation. Some 100 μL of enzyme-labeled antibody was added to each well, the membrane was added, and they were incubated at room temperature in the dark for 30 min. Similarly, after discarding the liquid, they were washed with washing solution four times, and the plates were dried after each operation. Briefly, 100 μL of TMB substrate solution was added to each well and incubated at room temperature in the dark for 10 min; then, 100 μL of stop solution was added to each well to stop the reaction. The optical density value (OD value) of each well was measured at a wavelength of 450 nm using a microplate reader.

### 2.2. Database Construction of Active Ingredients and Potential Targets

The chemical ingredients of MFEC were obtained from the Traditional Chinese Medicine Database and Analysis Platform (TCMSP) (https://old.tcmsp-e.com/tcmsp.php) (accessed on 16 April 2014). The active compounds were selected according to pharmacokinetic parameters including absorption, distribution, metabolism, and excretion. Basically, the criteria for the related parameters were set follows: oral bioavailability (OB) not less than 30%, and drug-likeness (DL) not less than 0.18.

The potential targets of the active ingredients were obtained from TCMSP, TCM-ID (http://119.3.41.228:8000/tcmid/) (accessed on 16 April 2014), PubChem (https://pubchem.ncbi.nlm.nih.gov/) (accessed on 16 April 2014) and the Swiss Target Prediction database (http://www.swisstargetprediction.ch/) (accessed on 16 April 2014). Additionally, all the targets were integrated, and duplicates were removed.

### 2.3. Acquisition and Screening of Immunosuppression-Associated Targets

The GeneCards database (https://www.genecards.org/) (accessed on 16 April 2014) was used to extract the immunosuppression-related targets, and the median value of relevance score was used to screen the obtained targets. Only the targets with an inference score no less than 9.33 were included. Besides, the Uniprot database (https://www.uniprot.org/) (accessed on 16 April 2014) was used to convert the obtained disease-related targets and the potential targets of the active ingredients obtained in Section 2.2 into the gene symbol formats. By merging all acquired genes, all targets related to immunosuppression were collected to establish a gene library of anti-immunosuppression targets. The Venny 2.1 tool (https://bioinfogp.cnb.csic.es/tools/venny/) (accessed on 16 April 2014) was used to determine the number of intersection genes between MFEC potential targets and disease-related genes, and to plot a Venn diagram.

### 2.4. Network Construction and Topological Analysis

The network’s construction and topological analysis were investigated using String 11.5 database (https://string-db.org/) (accessed on 16 April 2014) and Cytoscape 3.8.1 software. An intersection ingredient–target network was constructed to understand the associations between the active ingredients and intersection targets of MFEC. Network generation and visualization were performed using Cytoscape software 3.8.1. In order to construct a PPI network, common targets were imported into the String 11.5 database to generate a network, and the topological analysis was conducted using Cytoscape software, wherein the degree centrality, closeness centrality, and the betweenness centrality were obtained to evaluate the central properties of the nodes in the network.

### 2.5. GO and KEGG Pathway Enrichment Analysis

To further elucidate the pharmacological mechanisms of MFEC in immunosuppression treatment, Metascape database (https://metascape.org/) (accessed on 18 December 2021), a web-based portal that covers a comprehensive list of gene annotations, was used to perform a Gene Ontology (GO) enrichment analysis and Kyoto Encyclopedia of Genes and Genomes (KEGG) pathway enrichment analysis. The results were analyzed and sorted according to the adjusted *p* value. Additionally, the target–pathway network was established using Cytoscape software 3.8.1.

### 2.6. Molecular Docking Verification

The molecular docking was performed to predict the binding affinity and interactions between the targets with the active ingredients. Based on the topological parameters of the ingredient–target and PPI networks, ingredients and targets with more than 2-fold the median value of the degree were selected as candidate ligands and receptors for molecular docking. The software ChemBio3D 19.0 was used to calculate the minimizing energy, and the ligand structures were exported in MOL 2 format. ChemBio3D has been widely used for the simulation and analysis of the protein receptor–ligand small molecule complex. It is capable of calculating molecular energy-minimizing configurations, carrying out the structural optimization of model molecules, and simulating analyses to examine the stable structures of molecules [14,15]. The 3D structures of selected receptors were screened and downloaded from the RCSB PDB database (https://www.rcsb.org/) (accessed on 10 November 2022). The Sybyl-x 2.1.1 software could perform the molecular docking of active compounds with putative targets. All the pieces of software and databases used in this study are described in Supplementary Information Table S4.

### 2.7. Molecular Dynamics Simulations

The above part of molecular docking results (arachidonic acid-HSP90AA1; Dehydrodieugenol-HSP90AA1; icosa-11,14,17-trienoic acid methyl ester-HSP90AA1; (-)-tabernmontanine-AR; beta-carotene-AR; beta-sitosterol-AR) were performed using Gromacs 2020.6 software for molecular dynamics simulations. The simulation system was adjusted to a sodium chloride solution at 37 °C in order to replicate the actual human in vivo environment. Considering the active ingredients of MF and EC as multi-carbon ring skeleton structures, a Charmm36 force field and TIP3P water model were chosen for MDs. During the MDs, the relevant hydrogen bonds are constrained by the Linear Constraint Solver (LINCS) algorithm with the integration step of 2 fs, while the non-bond interaction cut-off value is set to 10 Å, and updated every 10 steps. Additionally, the electrostatic interactions are calculated using the particle-mesh Ewald (PME) with a cut-off value of 1.2 nm. In order to optimize the original conformation of the protein in the solvent, the protein receptor–ligand small molecule complex was pre-equilibrated for 100 ps prior to simulations, followed by canonical ensemble (NVT) equilibration for 100 ps using a modified Berendsen temperature coupling algorithm with a coupling time constant of 0.1 ps, which allowed the complex to be warmed up to 310 K with the solvent system. Subsequently, the solvent and complex were pressure equilibrated, while the pressure was increased to 1 bar using a Berendsen constant pressure, with constant-pressure and constant-temperature (NPT) equilibration of 100 ps. Finally, MDs of the complex were performed for 50 ns.

## 3. Results

### 3.1. Mice Spleen Index, IL-2, IL-6, and TNF-α Level in Serum

**Figure 1:**
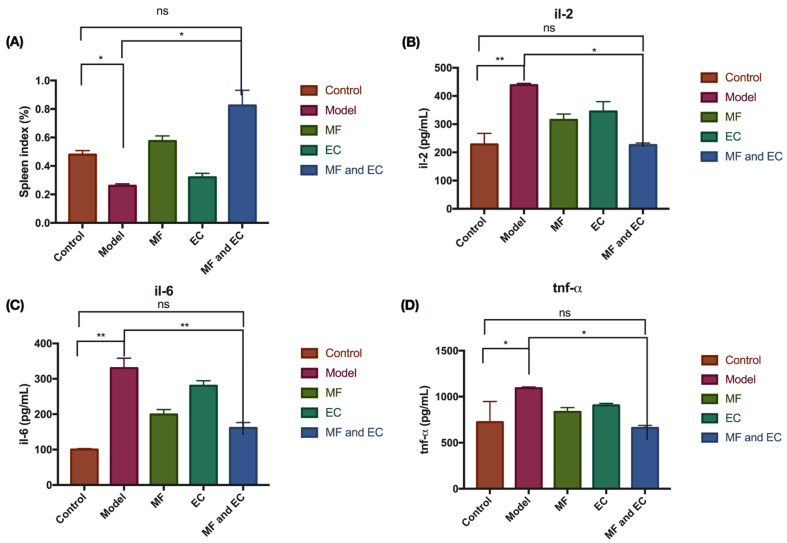
TMF and EC inhibit cyclophosphamide-induced inflammation in mice. (**A**) The spleen index of mice for control group, inflammation group, and treatment with MF, or EC, or MF and EC. (**B**–**D**) The expression level of IL-2, IL-6, and TNF-α. Compared with control group, the spleen index in the model group decreased, whereas the expression level of pro-inflammatory factors IL-2, IL-6, and TNF-α increased in the model group. (* *p* > 0.1, ** *p* > 0.01, ns stands for no significant difference; *t*-test and ANOVA analysis).

It can be seen from Figure 1 that compared with the control group, the spleen index of the mice in the model group decreased. After treatment with MF, EC and MFEC, the spleen index of the mice increased compared with the model group, and the spleen index of the MFEC group increased more obviously; however, the statistical analysis showed no significant difference. Comparing the levels of TNF-α in all groups, the content of TNF-α in the MF group was slightly lower than that in the control group, but there was no significant change in the other groups. Taken together, these data indicate that the drug may have a certain therapeutic effect on the immunosuppression of mice.

### 3.2. Database Construction of Active Ingredients and Potential Targets

Based on the criteria of the OB ≥ 30% and DL ≥ 0.18, a total of 53 active ingredients (25 in MF, 24 in EC, 4 common ingredients, as shown in Supplementary Materials Table S1) were retrieved from the TCMSP database. All the potential targets of the active ingredients were found in the TCMSP, TCM-ID, Swiss Target Prediction and Pubchem databases. After removing duplicates, 423 potential targets were screened for in the two traditional Chinese medicines.

### 3.3. Immunosuppression-Related Targets

**Figure 2:**
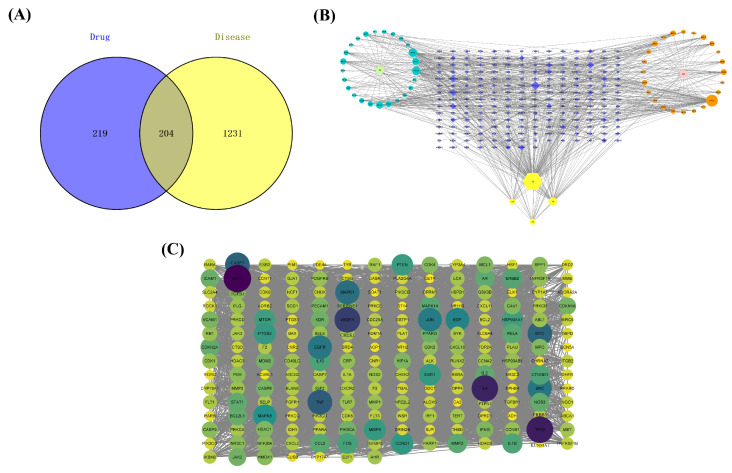
Construction of database and network. (**A**) Venn diagram of MFEC targets and immune suppression disease gene set. (**B**) Network of ingredient–target interaction: the blue dots represent MF compounds, the light green represents the drug MF, the orange dots are EC compounds, the pink dots are EC drugs, the yellow hexagonal nodes are the common compounds of MFEC, and the blue diamonds are the intersection targets of drugs and diseases. (**C**) PPI network.

The immunosuppression-associated targets were retrieved from the GeneCards database. As a result, a total of 11,461 disease-related genes were obtained. Based on three screenings of the median value, 1435 disease-related genes were selected. Furthermore, 204 common targets (in Supplementary Materials Table S2) between MFEC targets and disease-related targets were filtered as the critical targets for further study (Figure 2A).

### 3.4. Compound—Target Interaction Network

The compound–target interaction network is visualized in Figure 2B with 259 nodes and 665 edges by Cytoscape. In the network, nodes represent the screened drug, active ingredients, and genes, while the connections between the nodes represent the interactions between these biological analyses. In the current study, active compounds with a degree 2-fold higher than the median value were selected to perform molecular docking. These 11 active compounds are shown in Supplementary Materials Table S3.

### 3.5. PPI Network

To analyze the protein–protein interaction, the 204 common targets of ingredients and diseases were imported into the String database and Cytoscape to show complex interaction between proteins encoded by these targets. The PPI network covered 203 nodes (the protein IGHG1 was not identified) and 4897 edges (Figure 2C). Among the 203 proteins, AKT1, AR, CASP3, HSP90AA1, JUN, MAPK14, MMP2, PTSG2, and TNF were hub proteins finally selected as putative targets to conduct molecular docking.

### 3.6. GO and KEGG Enrichment Analysis

#### 3.6.1. GO Enrichment

**Figure 3:**
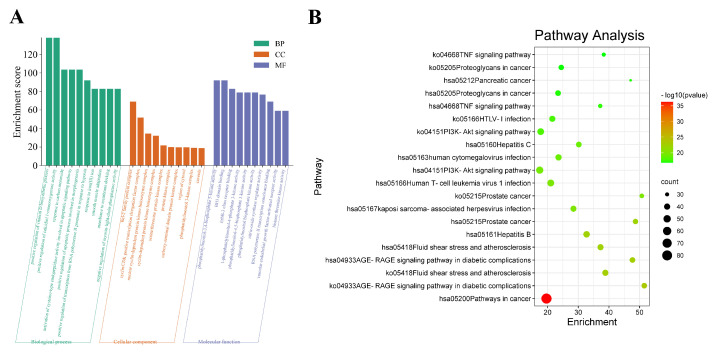
GO and KEGG enrichment analysis. (**A**) GO enrichment. The green bars represent the analysis of BP, the orange represent the analysis of CC, and the blue represent the analysis of molecular function; (**B**) Bubble diagram of KEGG pathways. The horizontal axis represents the gene enrichment, while the longitudinal axis represents pathway terms. The bubble’s color represents the significance level (*p*-value) of the corresponding pathways: the significance level decreases (*p*-value increases) from red to green. Additionally, the bubble’s size represents the gene count of the pathway.

GO enrichment analysis was performed using Metascape software version v3.5.20230501 on 204 common targets of immunosuppression and Mori Folium and Eucommiae Cortex in terms of biological process (BP), cellular composition (CC), and molecular function [13]. As seen in Figure 3A, the biological process mainly included positive regulation of the vitamin D biosynthetic process, positive regulation of calcidiol 1-monooxygenase activity, response to carbon monoxide, activation of cysteine-type endopeptidase activity involved in the apoptotic signaling pathway, and positive regulation of the apoptotic process involved in morphogenesis. The cellular composition was mainly enriched by the Bcl-2 family protein complex, cyclin/CDK positive transcription elongation factor complex, and the nuclear cyclin-dependent protein kinase holoenzyme complex. The molecular function mainly involved phosphatidylinositol-3,4-bisphosphate 5-kinase activity, BH3 domain binding, ErbB-3 class receptor binding, 1-phosphatidylinositol-4-phosphate 3-kinase activity, and phosphatidylinositol-4,5-bisphosphate 3-kinase activity.

#### 3.6.2. KEGG Enrichment

KEGG pathway analyses can indicate possible signaling pathways of 204 proteins, and there were 362 pathways obtained in total. Figure 3B and Supplementary Material Table S5 show specific information about the underlying mechanisms involved in MFEC to treat immune suppression. The top three enriched KEGG pathways were pathways in cancer, and the AGE-RAGE signaling pathway in diabetic complications, fluid shear stress, and atherosclerosis.

### 3.7. Molecular Docking

**Figure 4:**
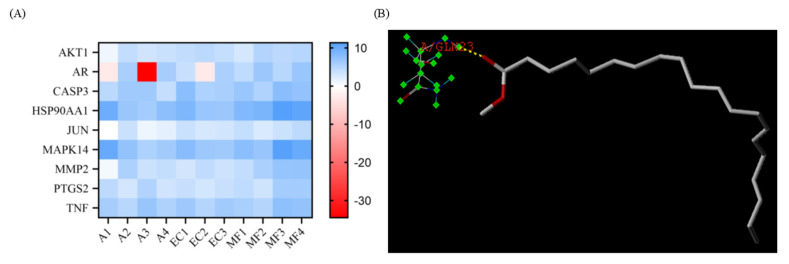
Molecular docking. (**A**) Heat map of total score between compounds and proteins. (**B**) The structure of the best-ranked pose in molecular docking: docking result of HSP90AA1 with icosa-11,14,17-trienoic acid methyl ester.

Some nine proteins were selected according to the PPI network, and the structures of these nine proteins were downloaded from the PDB database. Sybyl-X software version 2.2.1 was used to remove water and excess ligands in these nine proteins, and then they were hydrogenated. The last step in pre-processing the protein was to generate binding pockets. These nine proteins were docked with eleven active components the related results are shown in Table 1, Figure 4A,B, and Supplementary Information Figure S1. In Table 1, we can see that among all 11 active compounds, iristectorigenin A, quercetin, icosa-11,14,17-trienoic acid methyl ester, and arachidonic acid might be important active compounds. Among all nine protein targets, iristectorigenin A had better docking results with AKT1 and AR, and there were three docking sites with each target. Quercetin and CASP3 docking have five binding sites, which is the most in all docking. Icosa-11,14,17-trienoic acid methyl ester binds to HSP90AA1 and TNF in a docking site, respectively. Arachidonic acid has the best docking effect with four of the screened targets and has a high score. In addition to two binding sites with PTGS2, arachidonic acid has one docking site with JUN, MAPK14 and MMP2. The structure of the best-ranked is in Figure 4B, and all other poses during molecular docking are presented in Figure S1.

### 3.8. Molecular Dynamics Simulations

**Figure 5:**
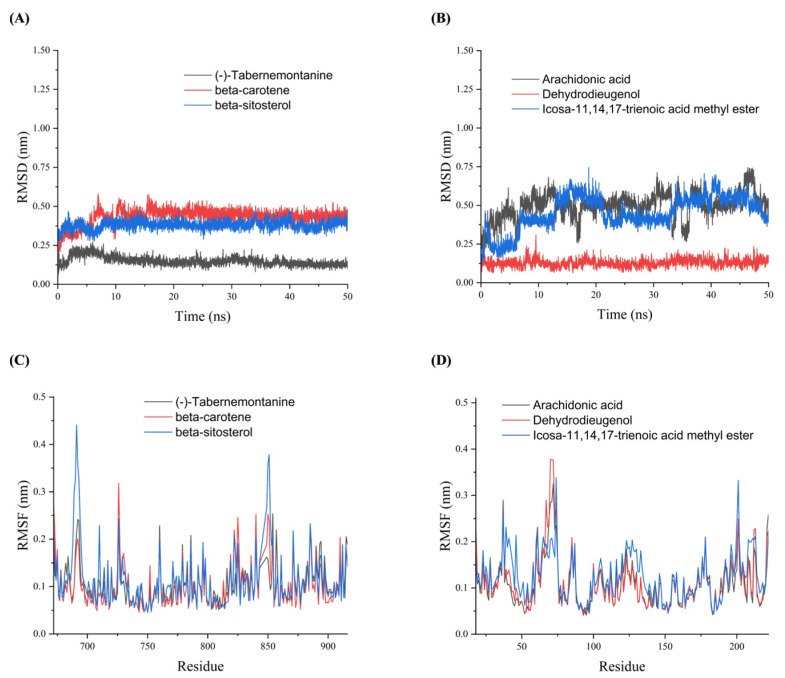
The RMSD and RMSF plots of molecular dynamics simulations. (**A**) RMSD plots of AR with (-)-tabernemontanine, beta-carotene and beta-sitosterol. (**B**) RMSD plots of HSP90AA1 with arachidonic acid, Dehydrodieugenol, and icosa-11,14,17-trienoic acid methyl ester. (**C**) RMSF plots of AR with (-)-tabernemontanine, beta-carotene and beta-sitosterol. (**D**) RMSF plots of HSP90AA1 with arachidonic acid, Dehydrodieugenol, and icosa-11,14,17-trienoic acid methyl ester.

To further validate the binding stability of drug active ingredients with AR and HSP90AA1 in vivo, analyses of root mean square deviation (RMSD), root mean square fluctuation (RMSF), radius of gyration (Rg) and hydrogen bond (Hbond) were obtained using Gromacs 2020.6 software. RMSD was used to analyze the binding stability of the AR and HSP90AA1 to the corresponding ligand receptor in the MDs. Figure 5A shows that the (-)-tabernemontanine-AR complex reached dynamic equilibrium within a short time (10 ns), and the RMSD value remained around 0.12 nm until the end of the simulation, indicating that AR bound well with (-)-tabernemontanine and can form a stable complex. Meanwhile, the binding of AR with beta-sitosterol and beta-carotene reached an equilibrium at 10–15 ns, and its RMSD fluctuation value was less than 0.3 nm, which suggested stable drug–protein complex binding. Figure 5B suggested that arachidonic acid-HSP90AA1 and icosa-11,14,17-trienoic acid methyl este-HSP90AA1 always had large fluctuations in the RSMD curves during the MDs, indicating that the active ingredient of the drug could not bind stably to the protein target. RMSF was used to reflect the fluctuations and the degree of motion drasticity of protein residues throughout the MDs. Figure 5C,D showed that AR and HSP90AA1 residues had greater flexibility and adaptability when key drug active ingredients were bound to hub protein target. 

**Figure 6:**
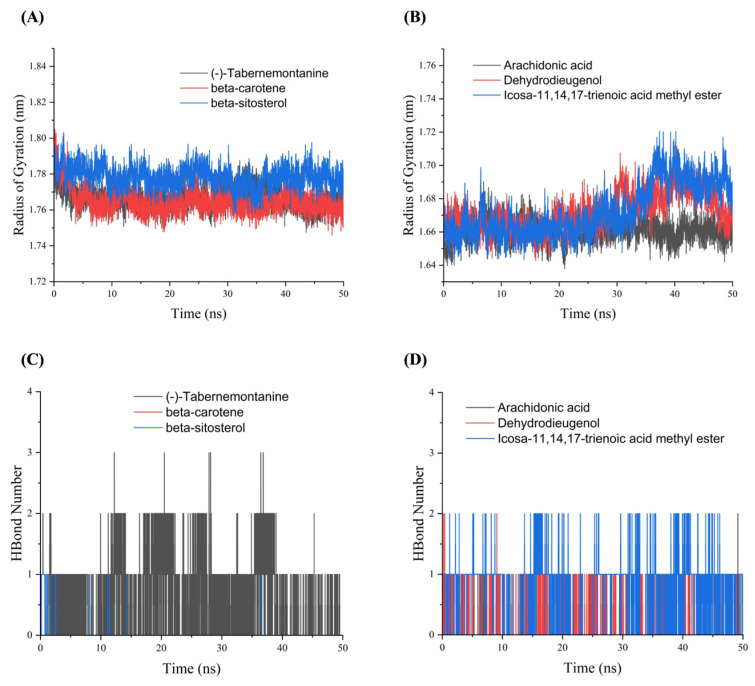
The Rg and Hbond plots of molecular dynamics simulation. (**A**) Rg plots of AR with (-)-tabernemontanine, beta-carotene and beta-sitosterol. (**B**) Rg plots of HSP90AA1 with arachidonic acid, Dehydrodieugenol, icosa-11,14,17-trienoic acid methyl ester. (**C**) Hbond plots of AR with (-)-tabernemontanine, beta-carotene and beta-sitosterol. (**D**) Hbond plots of HSP90AA1 with arachidonic acid, Dehydrodieugenol, icosa-11,14,17-trienoic acid methyl ester.

Rg indicated the tightness of the overall structure of the protein [14] and characterized changes in peptide chain relaxation of the protein during the simulation. Figure 6A indicates that the complexes formed by AR bounds to the corresponding three drug active molecules had a more stable radius of gyration, and gradually decreased in the MDs, which suggested that the proteins gradually converge and the complex structures become stable after binding to the small molecules. The Rg curves of HSP90AA1 showed some fluctuations (Figure 6B). As can be seen in Figure 6C, the beta-sitosterol-AR complex formed hydrogen bonds only in part of the time period, and the (-)-tabernemontanine-AR complex basically retained more than one hydrogen bond during the simulation. The interaction between beta-carotene and AR never formed a hydrogen bond (Figure 6C), because the beta-carotene-AR complex structure has no hydrogen bonds and the interaction be dominated by hydrophobic interactions. Figure 6D showed that the number of hydrogen bonds in the arachidonic acid-HSP90AA1 complex was low throughout the MDs. Figure 6D shows that the number of hydrogen bonds formed by the icosa-11,14,17-trienoic acid methyl ester-HSP90AA1 complex is 1–2. The dehydrodieugenol-HSP90AA1 complex maintained one hydrogen bond for most of the time throughout the MDs, which indicates that Dehydrodieugenol is more stable in binding to HSP90AA1 upon an RMSD analysis. 

## 4. Discussion

In recent decades, TCM has attracted worldwide attention due to its exact curative effects, relatively low toxicity, and low cost. Due to the complex components of TCM and the various biological systems in which they are involved, elucidating its mechanism of action has become a challenge [12,16]. Network pharmacology takes the main active ingredients and target proteins of TCM as nodes and uses edges to represent their interactions by generating an interaction network in order to elucidate the mechanism of action of TCM prescriptions at the molecular level [17].

In the present study, network pharmacology is used to explore the possibility of MFEC in the treatment of immunosuppression. The obtained relevant targets were used to dig out the putative related pathways, and to speculate on the possible mechanisms of MFEC treatment of immunosuppression. Additionally, molecular docking was used to verify the key components and targets of MFEC involved in the treatment of immunosuppression.

During the active component data-mining of MFEC, a total of 53 active components were collected. Finally, 11 critical components were selected according to the PPI and topological parameters. The 11 components were icosa-11,14,17-trienoic acid methyl ester, iristectorigenin A, arachidonic acid, tetramethoxyluteolin in molecular function; dehydrodieugenol, (-)-tabernemontanine, and (9R)-6′-methoxycinchonan-9-ol in EC, and their common active ingredients quercetin, kaempferol, beta-sitosterol, and beta-carotene. These 11 compounds mainly belong to flavonoids, alkaloids, phenolics, terpenes, phytosterols, and organic acids, which have pharmacological effects in MFEC. Both flavonoids and phenolic active ingredients in MFEC belong to polyphenolic compounds and contain a large number of aromatic hydroxyl groups, which account for their anti-inflammatory, anti-glycemic pharmacological effects, and prevent oxidative damage and cell death [11,18,19]. In addition, studies have shown that kaempferol can alleviate neuronal damage caused by kainic acid-induced epilepsy in mice, restore the proliferation of T lymphocytes to a certain extent, reduce the phagocytic function of macrophages, and inhibit the apoptosis of thymocytes [20]. Besides, kaempferol has certain immunomodulatory effects [20]. To explore the protective effect of organic acids on inflammatory injury in acute tracheobronchitis, LPS was used to establish respiratory inflammation in mice [21]. Results showed that organic acids could treat acute tracheobronchitis by regulating the TLR4/NF-κB signaling pathway, indicating that organic acids have certain anti-inflammatory effects [21].

The pharmacological effects of alkaloids are very extensive, and can be used for anticancer, antimalarial, antiviral, antihypertensive, antispasmodic, antiarrhythmic, analgesic, antibacterial, and antidiabetic purposes, and also as central nervous system stimulants and muscle relaxants, with vasodilatory properties [22]. The beta-sterols in MFEC belong to the class of phytosterols that have been shown to have cholesterol-lowering, anti-cancer, anti-atherosclerotic, anti-inflammatory, and antioxidant effects [23]. Beta-carotene belongs to the group of carotenoids in terpenoids, and has been proven to be the most abundant pigment and fat-soluble antioxidant in nature [24]. In addition, studies have demonstrated that the addition of fully oxidized beta-carotene to the feed can enhance the immunity and performance of sows [25].

Comprehensive analysis of GO enrichment results showed that the active components in MFEC may act on organisms through the nucleus, cytoplasm, membrane, cytosol, and organelle membrane; exert the molecular functions of phosphatidylinositol-3-kinase (PI3K) family protein kinases, BH3 domain binding, nitric oxide and other synthase regulators, vascular endothelial growth factor (VEGF) activator receptors, RNA polymerase 2 transcription coactivator, and so on; participate in the reaction of carbon monoxide and iron ions; positively regulate vitamin D biosynthesis, monooxygenase activity and cell apoptosis in vivo; and negatively regulate myosin light chain phosphatase and other series of biological processes so as to achieve the goal of therapeutic immunosuppression. The KEGG enrichment analysis showed that the main signaling pathways involved in the common targets of MFEC and immunosuppression included the pathway in cancer, advanced glycation end products and their receptors (AGEs-RAGE) signaling pathway in diabetic complications, PI3K-Akt signaling pathway, tumor necrosis factor (TNF) signaling pathway, fluid shear stress and atherosclerosis, kapos sarcoma-associated herpes virus infection, hepatitis B and C, and prostate and pancreatic cancer. AGEs are heterogeneous glycation products of proteins, lipids and nucleotides, and their receptors are called RAGE, which are multiligand transmembrane receptors of the immunoglobulin superfamily [26]. When the body’s redox homeostasis is disrupted, ROS accumulate, resulting in oxidative stress. Studies have shown that some active components in TCM could improve the level of intracellular oxidative stress, reduce the content of AGEs and ROS, and down-regulate the level of oxidative stress by inhibiting the AGEs-RAGE signaling pathway [27]. From the CC analysis, PI3K is a cytoplasmic lipid kinase, which is a heterodimer composed of a regulatory subunit p85 and a catalytic subunit p110. Additionally, the active PI3K phosphorylates to generate the second messenger phosphatidylinositol 3,4,5-triphosphate (PIP3), which further induces the phosphorylation of AKt, and thus participates in the regulation of various life processes such as growth, apoptosis, and oxidative stress [28]. TNF is one of the most well-studied cytokines in the immune system, which can regulate various processes such as cell communication, differentiation, and death. These regulatory functions are related to various diseases of the body, including the body’s autoimmunity [29]. 

The nine protein targets selected for molecular docking in this study were jointly selected by combining the analysis results of compound–target network and PPI network. AKT1, one of the AKT isoforms, is an oncogene that is ubiquitous in neurons as an important downstream substrate in the PI3K-Akt signaling pathway [30]. AKT1 also plays a key role in the normal development of the nervous system and memory formation when participating in the regulation of cell survival and growth [31]. When AKT1 is mutated, it can increase the risk of schizophrenia [32]. The androgen receptor (AR) is a ligand-activated nuclear receptor that interacts with the estrogen receptor (ER), glucocorticoid receptor (GR), progesterone receptor (PR), and mineralocorticoid receptor (MR); both belong to the type I nuclear receptor subfamily [33]. AR coordinates the expression of androgen-regulated transcriptomes in the nucleus, and is critical for prostate development, homeostasis, and carcinogenesis. Studies have shown that AR outside the nucleus can form a complex with Akt under androgen stimulation, and induce the phosphorylation and activation of Akt [34,35]. CASP3 is one of the final effector proteins in the apoptotic response. Tian et al. [36] have shown that under hypoxia and nutrient deprivation conditions, the activity of CASP3 in human-nucleus-pulposus-derived mesenchymal hepatocytes can be enhanced to promote cell apoptosis. HSP90AA1 is the α isoform of the heat shock protein (HSP) family with a molecular weight of 90 kDa. HSP90 has the function of maintaining protein stability and is involved in the arrangement and maintenance of almost the entire cytoskeleton [37]. Additionally, HSP90AA1 can promote autophagy through the PI3K/Akt/mTOR signaling pathway, and inhibit apoptosis through the JNK/P38 signaling pathway to improve the drug resistance of osteosarcoma cells [18]. JUN is an important component of the transcriptional activation protein complex AP1 (activation protein-1, AP1), which is widely involved in many biological processes such as cell proliferation, differentiation and apoptosis. JUN can form dimers by combining with other components of the AP1 complex to jointly regulate the expression of downstream target genes [37]. MAPK14 belongs to the mitogen-activated protein kinase (MAPK) family of proteins, enhancing the formation of tumor platelet aggregates that interact with lung endothelium to form lung metastases [38]. MMP2 belongs to the family of matrix metalloproteinases (MMPs), which are known as inflammatory mediators [39]. In the process of atherosclerosis (AS), MMP2 can degrade a variety of collagen and basement membrane components, lyse the collagen fibers of AS plaques, and reduce the thickness of the fibrous cap at the plaque. As a highly important evaluation factor [40], PTGS2, also known as cyclooxygenase 2 (COX-2), is a pro-inflammatory factor that is normally not expressed in most tissues. Its expression is induced by a variety of stimuli, such as epidermal growth factor (EGF), interleukin-1 (IL-1), and TNF, and inflammatory responses may also induce the production of PTGS2 [41,42]. TNF mainly refers to TNF-α, as the first inflammatory factor in the inflammatory response, TNF can activate lymphocytes and neutrophils, and play a crucial role in initiating and expanding the inflammatory cascade [43].

Molecular docking revealed drug–target and protein–target interactions from the molecular perspective, and MDs have been widely used to study the binding stability between proteins and molecules and evaluate the structural features of protein–ligand systems [44]. Therefore, in this study, we adopted a combination of molecular docking and molecular dynamics simulation to screen the key targets of ML and EC for enhancing immune function and verifying the stability of active ingredients binding to protein targets. During molecular docking using Autodock, a score function, which is composed of linear weights and a force field, will be used to obtain the binding energy. The available score functions include the Vinardo scoring function, empirical scoring function, Dk_scoring function, etc. Vinardo is a novel approach that is freely available from the link http://smina.sf.net (accessed on 18 November 2021). Vina, iDock, and Smina utilize an empirical scoring function. Dk_scoring function refers to the linear regression between the calculated and experimental binding energies. The molecular docking results suggests that iristectorigenin A, quercetin, acid methyl ester, arachidonic acid exhibited great binding affinity to nine hub targets of immunosuppression. From the analysis of the results of RMSD, RMSF, RG and Hbond of MDs, it is clear that the (-)-tabernemontanine-AR, beta-sitosterol-AR, and Dehydrodieugenol-HSP90AA1 complexes bind stably. Molecular docking and further MDs’ validation finally identified AR, MAPK14, TNF, and HSP90AA1 as the core targets for immunosuppression.

## 5. Conclusions

The effects and pharmacological mechanisms of Mori Folium and Eucommiae Cortex (MFEC) extracts against immunosuppression were deciphered by integrating network pharmacology analysis, molecular docking, molecular dynamics simulation, and in vivo validation. The results indicated MFEC exerted pharmacological efficacy via multiple targets and multiple pahways. This study provides a theoretical basis for in-depth exploration of the pharmacological effects of MFEC and its mechanism of action in the treatment of immunosuppression. 

## Figures and Tables

**Table 1:** Molecular docking results.

Protein	Compound	Binding Site	Total Score
AKT1	iristectorigenin A	A/SER56, A/LEU110, A/GLN59	5.0365
AR	iristectorigenin A	A/ASN705, A/GLN711, A/MET745	6.5950
CASP3	quercetin	A/ARG64, A/SER205, A/GLY165, A/ARG164, A/GLU123	6.2159
HSP90AA1	icosa-11,14,17-trienoic acid methyl ester	A/GLN23	11.3399
JUN	arachidonic acid	B/MET253	4.3453
MAPK14	arachidonic acid	A/HIS148	10.0545
MMP2	arachidonic acid	A/ASN573	7.0050
PTGS2	arachidonic acid	B/GLU486, B/ARG438	6.0676
TNF	icosa-11,14,17-trienoic acid methyl ester	D/TYR151	7.4725

## Data Availability

All data are contained within the manuscript or in Supplementary Materials.

## Supplementary Materials

The following supporting information can be downloaded at: https://www.mdpi.com/article/10.3390/metabo13111151/s1, Table S1: Detailed information on active compounds; Table S2: The 204 common targets (Table S3) between MFEC targets and disease-related targets; Table S3: The information of the active ingredients, including their 2D structures; Table S4: The pieces of software and databases used in this study; Table S5: KEGG pathway analysis of MFEC used to treat immune suppression; Figure S1: The structures of other poses in molecular docking.
