# Supplementary material for: The Mechanism of Mori Folium and Eucommiae Cortex against Cyclophosphamide-Induced Immunosuppression Integrating Network Pharmacology, Molecular Docking, Molecular Dynamics Simulations, and Experimental Validation
Source: Metabolites. 2023 Nov 15;13(11):1151. doi: 10.3390/metabo13111151 (PMC10673040; doi:10.3390/metabo13111151)
Supplement: Supplementary file 1 [file metabolites-13-01151-s001.zip › metabolites-2660937-supplementary.pdf]

Supplementary Material

1 Supplementary Figures and Tables

1.1 Supplementary figures

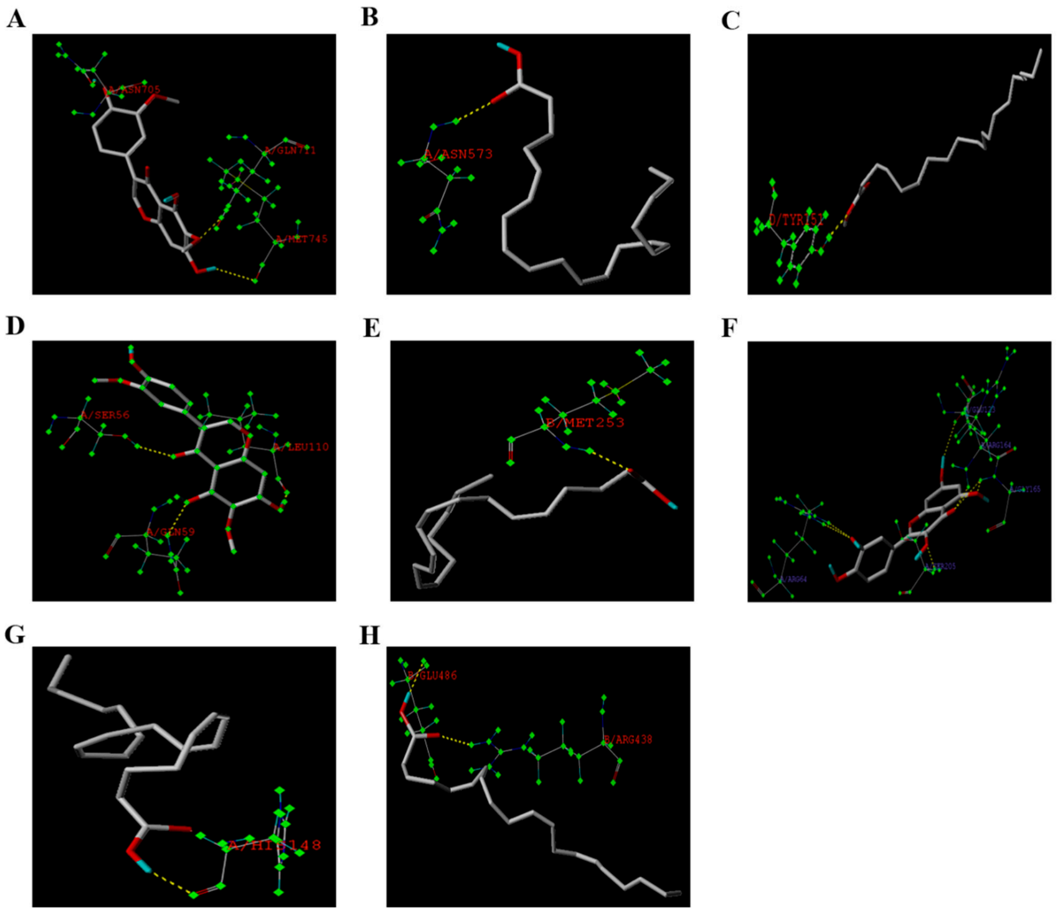

Figure S1. The structures of other poses in molecular docking. The binding site of molecular docking: docking results of AR(A) and AKT1(D) with iristectorigenin A; docking results of MMP2(B), JUN(E) MAPK14(G) and PTSG2(H) with arachidonic acid; docking results of TNF(C) with icosa-11,14,17-trienoic acid methyl ester; docking results of CASP3 (F) with quercetin.

1.2 Supplementary tables

Table S1. Detailed information on active compounds.

ID	MOL ID	Name	OB	DL	Inchi Key	Herb
A1	MOL002773	beta-carotene	37.18	0.58	OENHQHLEOONYIE-JLTXGRSLSA-N	MF, EC
A2	MOL000098	quercetin	46.43	0.28	REFJWTPEDVJJIY-UHFFFAOYSA-N	MF, EC
A3	MOL000358	beta-sitosterol	36.91	0.75	KZJWDPNRJALLNS-VJSFXXLFSA-N	MF, EC
A4	MOL000422	kaempferol	41.88	0.24	IYRMWMYZSQPJKC-UHFFFAOYSA-N	MF, EC
EC1	MOL009030	Dehydrodieugenol	30.1	0.24	KETPSFSOGFKJJY-UHFFFAOYSA-N	EC
EC2	MOL009015	(-)-Tabernemontanine	58.67	0.61	FFVRRQMGGGTQRH-MSPRAJMNSA-N	EC
EC3	MOL009031	(9R)- 6'- methoxycinchonan-9-ol	68.22	0.4	LOUPRKONTZGTKE-AFHBHXEDSA-N	EC
EC4	MOL000073	ent-Epicatechin	48.96	0.24	PFTAWBLQPZVEMU-ZFWWWQNUSA-N	EC
EC5	MOL011604	Syringetin	36.82	0.37	UZMAPBJVXOGOFT-UHFFFAOYSA-N	EC
EC6	MOL000211	Mairin	55.38	0.78	QGJZLNKBHJESQX-FZFNOLFKSA-N	EC
EC7	MOL009055	hirsutin_qt	49.81	0.37	UIZYKATWALCYCD-UHFFFAOYSA-N	EC
EC8	MOL009057	liriodendrin_qt	53.14	0.8	KJHRHHLEZJFLDH-OZCSAQGGSA-N	EC
EC9	MOL000443	Erythraline	49.18	0.55	TVOFUERNMZTYRM-	EC

					KBXCAEBGSA-N	
EC10	MOL002058	Medioresinol	57.2	0.62	VJOBNGRIBLNUKN-BMHXQBNDSA-N	EC
EC11	MOL009053	4-[(2S,3R)-5-[(E)-3-hydroxyprop-1-enyl]-7-methoxy-3-methylol-2,3-dihydrobenzofuran-2-yl]-2-methoxy-phenol	50.76	0.39	KUSXBOZNRPQEON-LNFBDUAVSA-N	EC
EC12	MOL004367	olivil	62.23	0.41	BVHIKUCXNBQDEM-XMCHAPAWSA-N	EC
EC13	MOL005922	Acanthoside B	43.35	0.77	WEKCEGQSIIQPAQ-IRBNZIFYSA-N	EC
EC14	MOL006709	8-Hydroxypinoresinol	92.43	0.55	CICMVLOHBZPXIT-WNISUXOKSA-N	EC
EC15	MOL007059	3-beta-Hydroxymethyllenetanshiquinone	32.16	0.41	RUJKJFRMCYQMLH-ZDUSSCGKSA-N	EC
EC16	MOL007563	Yangambin	57.53	0.81	HRLFUIXSXUASEX-RZTYQLBFSA-N	EC
EC17	MOL008240	(E)-3-[4-[(1R,2R)-2-hydroxy-2-(4-hydroxy-3-methoxy-phenyl)-1-methylol-ethoxy]-3-methoxy-phenyl]acrolein	56.32	0.36	LWLOALZBDOVWAE-FMEUAVTJSA-N	EC
EC18	MOL009007	Eucommin A	30.51	0.85	GLGVEKKQPFRBAS-UOVCOODASA-N	EC
EC19	MOL009009	(+)-medioresinol	87.19	0.62	VJOBNGRIBLNUKN-HANNOOJCSA-N	EC
EC20	MOL009038	GBGB	45.58	0.83	FYZYXYLPBWLLGI-AUOPOVQUSA-N	EC
EC21	MOL009027	Cyclopamine	55.42	0.82	QASFUMOKHFSJGL-LAFRSMQTSA-N	EC
EC22	MOL009029	Dehydrodiconiferyl alcohol 4,gamma'-di-O-beta-D-glucopyanoside_qt	51.44	0.4	GQNBCFQSSDOEHP-LNFBDUAVSA-N	EC
EC23	MOL009042	Helenalin	77.01	0.19	ZVLOPMNVFLSSAA-	EC

					XEPQRQSNSA-N	
EC24	MOL009047	(+)-Eudesmin	33.29	0.62	PEUUVVGQIVMSAW-RZTYQLBFSA-N	EC
MF1	MOL007879	Tetramethoxyluteolin	43.68	0.37	CLXVBVLQKLQNRQ-UHFFFAOYSA-N	MF
MF2	MOL003759	Iristectorigenin A	63.36	0.34	CCRPIWFQMLICCY-UHFFFAOYSA-N	MF
MF3	MOL003975	icosa-11,14,17-trienoic acid methyl ester	44.81	0.23	XQAVRBUXEPJVRC-JSIPCRQOSA-N	MF
MF4	MOL001439	arachidonic acid	45.57	0.2	YZXBAPSDXZZRGB-DOFZRALJSA-N	MF
MF5	MOL000433	FA	68.96	0.71	OVBPIULPVIDEAO-LBPRGKRZSA-N	MF
MF6	MOL000449	Stigmasterol	43.83	0.76	HCXVJBMSMIARIN-PHZDYDNGSA-N	MF
MF7	MOL000729	Oxysanguinarine	46.97	0.87	UFHGABBBZRPRJV-UHFFFAOYSA-N	MF
MF8	MOL001506	Supraene	33.55	0.42	YYGNTYWPHWGJRM-AAJYLUCBSA-N	MF
MF9	MOL001771	poriferast-5-en-3beta-ol	36.91	0.75	KZJWDPNRJALLNS-FBZNIEFRSA-N	MF
MF10	MOL002218	scopolin	56.45	0.39	SGTCGCCQZOUMJJ-YMILTQATSA-N	MF
MF11	MOL003842	Albanol	83.16	0.24	MJJWBJFYYRAYKU-FJPSCYHJSA-N	MF
MF12	MOL003847	Inophyllum E	38.81	0.85	YRHQANFINIANSK-UHFFFAOYSA-N	MF
MF13	MOL003850	26-Hydroxy-dammara-20,24-dien-3-one	44.41	0.79	GQNLRMVMODTKOO-	MF

					YUICVKHOSA-N	
MF14	MOL003851	Isoramanone	39.97	0.51	NWFNMRFBJUONKD-YJQDOJAJSA-N	MF
MF15	MOL003856	Moracin B	55.85	0.23	GOUSNRMGQRTROZ-UHFFFAOYSA-N	MF
MF16	MOL003857	Moracin C	82.13	0.29	ZTGHWUWBQNCCOH-UHFFFAOYSA-N	MF
MF17	MOL003858	Moracin D	60.93	0.38	CHAAQDMHLLQJRO-UHFFFAOYSA-N	MF
MF18	MOL003859	Moracin E	56.08	0.38	GDSYWTBPYYYLLE-UHFFFAOYSA-N	MF
MF19	MOL003860	Moracin F	53.81	0.23	MQRQKQBOYQLFAI-UHFFFAOYSA-N	MF
MF20	MOL003861	Moracin G	75.78	0.42	PRQYZCKJWCQXNM-UHFFFAOYSA-N	MF
MF21	MOL003862	Moracin H	74.35	0.51	QRLJHLHVDMQXPO-UHFFFAOYSA-N	MF
MF22	MOL003879	4-Prenylresveratrol	40.54	0.21	WWFOQQIWOKJBSJ-PLNGDYQASA-N	MF
MF23	MOL006630	Norartocarpetin	54.93	0.24	ZSYPIPFQOQGYHH-UHFFFAOYSA-N	MF
MF24	MOL007179	Linolenic acid ethyl ester	46.1	0.2	JYYFMIOPGOFNPK-AGRJPVHOSA-N	MF
MF25	MOL013083	Skimmin (8CI)	38.35	0.32	VPAOSFFTKWUGAD-TVKJYDDYSA-N	MF

Table S2. 204 common targets (Table S3) between MFEC targets and disease-related targets.

Target	Gene symbol	Compound
ATP-binding cassette sub-family A member 1	ABCA1	MF4

ATP-binding cassette sub-family G member 2	ABCG2	A2
Tyrosine-protein kinase ABL	ABL1	EC1
Adenosine A2a receptor	ADORA2A	EC1, EC20, MF3
Beta-2 adrenergic receptor	ADRB2	A2, A3, EC2, EC3, EC6, EC15, EC24, MF6, MF22
Aryl hydrocarbon receptor	AHR	A2, A4
RAC-alpha serine/threonine-protein kinase	AKT1	A1, A2, A4
ALK tyrosine kinase receptor	ALK	EC1
Arachidonate 5-lipoxygenase	ALOX5	A2, A4, EC1, MF3, MF4
Androgen receptor	AR	A2, A4, EC2, EC5, EC21, MF1, MF2, MF12, MF13, MF15, MF17, MF18, MF23
Apoptosis regulator BAX	BAX	A2, A3, A4
Apoptosis regulator Bcl-2	BCL2	A1, A2, A3, A4, MF3, MF11
Bcl-2-like protein 1	BCL2L1	A2, MF11
Baculoviral IAP repeat-containing protein 5	BIRC5	A2
Bromodomain-containing protein 4	BRD4	EC1, MF13
Carbonic anhydrase II	CA2	EC7, EC15, EC20, MF25
Caspase-3	CASP3	A1, A2, A3, A4, MF4, MF5
Caspase-7	CASP7	A1
Caspase-8	CASP8	A1, A2, A3
Caspase-9	CASP9	A1, A2, A3
Calcium sensing receptor	CASR	MF3
Caveolin-1	CAV1	A1, A2
C-C motif chemokine 2	CCL2	A2
Cyclin-A2	CCNA2	EC1, EC5, EC17, EC22, MF2, MF16, MF22
G2/mitotic-specific cyclin-B1	CCNB1	A2, EC1
G1/S-specific cyclin-D1	CCND1	A2, MF4
Cyclin T1	CCNT1	EC1
CD40 ligand	CD40LG	A2
Dual specificity phosphatase Cdc25A	CDC25A	MF13
Cell division control protein 2 homolog	CDK1	A2, A4, EC1, MF3
Cell division protein kinase 2	CDK2	EC1, EC5, EC17, MF1, MF2, MF17, MF18, MF20, MF22
Cell division protein kinase 4	CDK4	EC1, MF4
Cell division protein kinase 5	CDK5	EC1
Cell division protein kinase 9	CDK9	EC1
Cyclin-dependent kinase inhibitor 1	CDKN1A	A2
Cyclin-dependent kinase inhibitor 2A, isoforms 1/2/3	CDKN2A	A2
Cholesteryl ester transfer protein	CETP	MF4
Serine/threonine-protein kinase Chk1	CHEK1	MF1, MF2
Serine/threonine-protein kinase Chk2	CHEK2	A2
Neuronal acetylcholine receptor protein, alpha-7 chain	CHRNA7	A3, EC2, EC3, EC9, EC15, MF6
Inhibitor of nuclear factor kappa-B kinase subunit alpha	CHUK	A2
Cannabinoid receptor 1	CNR1	EC1, MF3
Cannabinoid receptor 2	CNR2	EC1, MF3, MF8
C-reactive protein	CRP	A2
Catenin beta-1	CTNNB1	A1
Cathepsin D	CTSD	A2
Cathepsin G	CTSG	EC1
C-X-C motif chemokine 10	CXCL10	A2

C-X-C motif chemokine 11	CXCL11	A2
C-X-C motif chemokine 2	CXCL2	A2
Interleukin-8 receptor B	CXCR2	EC1
Cytochrome P450 17A1	CYP17A1	MF13
Cytochrome P450 19A1	CYP19A1	EC18, MF3, MF11, MF13
Cytochrome P450 1A1	CYP1A1	A2, A4
Cytochrome P450 3A4	CYP3A4	A1, A2, A4
Dihydrofolate reductase	DHFR	MF5
Dipeptidyl peptidase IV	DPP4	A2, A4, EC5, EC11, EC15, EC17, EC22, MF1, MF2
D(2) dopamine receptor	DRD2	EC2, EC3
Transcription factor E2F1	E2F1	A2
Pro-epidermal growth factor	EGF	A2, MF4
Epidermal growth factor receptor	EGFR	A2, EC1, EC3
Leukocyte elastase	ELANE	EC1
ETS domain-containing protein Elk-1	ELK1	A2
Ephrin receptor	EPHB4	EC1
Receptor tyrosine-protein kinase erbB-2	ERBB2	A2, EC1
Receptor tyrosine-protein kinase erbB-3	ERBB3	A2
Estrogen receptor	ESR1	EC4, EC5, EC11, EC17, EC22, MF2, MF12, MF15, MF16, MF17, MF18, MF20, MF21, MF22
Estrogen receptor beta	ESR2	EC5, EC7, MF1, MF2, MF12, MF15, MF17, MF18
Thrombin	F2	MF11
Tissue factor	F3	A1, A2
Fibroblast growth factor receptor 1	FGFR1	EC1
Vascular endothelial growth factor receptor 1	FLT1	EC1
Tyrosine-protein kinase receptor FLT3	FLT3	EC1
Proto-oncogene c-Fos	FOS	A2
Glucose-6-phosphate 1-dehydrogenase	G6PD	MF4
Gap junction alpha-1 protein	GJA1	A1, A2
Glutamate [NMDA] receptor subunit epsilon 2	GRIN2B	MF3
Glycogen synthase kinase-3 beta	GSK3B	EC1, EC5, EC7, EC17, MF1, MF2, MF12, MF17, MF18, MF20, MF22
Glutathione S-transferase P	GSTP1	A2, A4
Beta-glucuronidase	GUSB	EC1
Histone deacetylase 1	HDAC1	EC1, MF5
Histone deacetylase 3	HDAC3	EC1
Histone deacetylase 6	HDAC6	MF5
Hypoxia-inducible factor 1-alpha	HIF1A	A2, EC13, EC18
Heme oxygenase 1	HMOX1	A1, A2, A4
Heat shock factor protein 1	HSF1	A2
Heat shock protein HSP 90-alpha	HSP90AA1	A2, A3, A4, EC2, EC3, EC4, EC5, EC7, EC10, EC11, EC12, EC15, EC17, EC19, EC22, EC24, MF1, MF2, MF15, MF16, MF17, MF18, MF19, MF23, MF25
Heat shock protein HSP 90-beta	HSP90AB1	A2, A3, A4, EC1, EC2, EC3, EC4, EC5, EC7, EC10, EC11, EC12, EC15, EC17, EC19, EC22, EC24, MF1, MF2, MF15, MF16, MF17, MF18, MF19, MF23, MF25
Heat shock protein beta-1	HSPB1	A2
Intercellular adhesion molecule 1	ICAM1	A2, A4, MF3

Isocitrate dehydrogenase [NADP] cytoplasmic	IDH1	MF3
Interferon gamma	IFNG	A2
Insulin-like growth factor II	IGF2	A2
Insulin-like growth factor-binding protein 3	IGFBP3	A2
Ig gamma-1 chain C region	IGHG1	EC7, EC15, MF6
Inhibitor of nuclear factor kappa-B kinase subunit beta	IKBKB	A4
Interleukin-10	IL10	A2
Interleukin-1 alpha	IL1A	A2
Interleukin-1 beta	IL1B	A2
Interleukin-2	IL2	A2
Interleukin-6	IL6	A2
Insulin receptor	INSR	A2, A4, EC1
Interferon regulatory factor 1	IRF1	A2
Integrin alpha-L	ITGAL	MF3
Integrin beta-2	ITGB2	MF3
Tyrosine-protein kinase JAK2	JAK2	EC1
Tyrosine-protein kinase JAK3	JAK3	EC1
Transcription factor AP-1	JUN	A1, A2, A3, A4
Calcium-activated potassium channel subunit alpha 1	KCNMA1	EC1, EC16, MF1
Lysine-specific histone demethylase 1	KDM1A	EC1
Vascular endothelial growth factor receptor 2	KDR	EC1, EC7, MF3
Tyrosine-protein kinase LCK	LCK	EC1
Mitogen-activated protein kinase 1	MAPK1	A2, MF4
Mitogen-activated protein kinase 14	MAPK14	EC5, EC7, EC17, MF1, MF2, MF13, MF17, MF18, MF20, MF22
Mitogen-activated protein kinase 8	MAPK8	A4
Induced myeloid leukemia cell differentiation protein Mcl-1	MCL1	EC13, EC18, MF3
p53-binding protein Mdm-2	MDM2	EC1
Hepatocyte growth factor receptor	MET	EC1
Neprilysin	MME	MF5
Interstitial collagenase	MMP1	A1, A2, A4, EC1
72 kDa type IV collagenase	MMP2	A1, A2, EC1
Stromelysin-1	MMP3	A2
Matrix metalloproteinase-9	MMP9	A2, EC1
Myeloperoxidase	MPO	A2
Serine/threonine-protein kinase mTOR	MTOR	EC1, MF11
Myc proto-oncogene protein	MYC	A1, A2
Neutrophil cytosol factor 1	NCF1	A2
Nuclear factor erythroid 2-related factor 2	NFE2L2	A2
NF-kappa-B inhibitor alpha	NFKBIA	A2
Nitric oxide synthase, inducible	NOS2	A4, EC5, EC7, MFMF1, MF2, MF13, MF17, MF18, MF21
Nitric oxide synthase, endothelial	NOS3	A2, MF4
NAD(P)H dehydrogenase [quinone] 1	NQO1	A2, EC1
LXR-alpha	NR1H3	MF3, MF13
Nuclear receptor subfamily 1 group I member 2	NR1I2	A2, A4
Glucocorticoid receptor	NR3C1	EC21, MF13
Mineralocorticoid receptor	NR3C2	EC21, MF6, MF14
Ornithine decarboxylase	ODC1	A2
Delta-type opioid receptor	OPRD1	EC2, EC3, EC9, EC15

Mu-type opioid receptor	OPRM1	A3, EC2, EC3, EC9, EC15
Poly [ADP-ribose] polymerase 1	PARP1	A2, EC1
Phosphodiesterase 4A	PDE4A	MF3
Platelet-derived growth factor receptor beta	PDGFRB	EC1
Platelet endothelial cell adhesion molecule	PECAM1	MF4
Progesterone receptor	PGR	A3, A4, EC6, MF6, MF9. MF14
PI3-kinase p110-alpha subunit	PIK3CA	EC1
PI3-kinase p110-beta subunit	PIK3CB	EC1
PI3-kinase p110-delta subunit	PIK3CD	EC1
PI3-kinase p110-gamma subunit	PIK3CG	A2, A3, A4, EC1, EC2, EC9, MF17
Proto-oncogene serine/threonine-protein kinase Pim-1	PIM1	EC1, EC5, EC11, EC17, EC22, MF2, MF12, MF22
Cytosolic phospholipase A2	PLA2G4A	MF4
Tissue-type plasminogen activator	PLAT	A2
Urokinase-type plasminogen activator	PLAU	A2, MF6, MF11
Plasminogen	PLG	MF11
Peroxisome proliferator-activated receptor alpha	PPARA	A2, MF3, MF8
Peroxisome proliferator-activated receptor delta	PPARD	A2, MF3
Peroxisome proliferator activated receptor gamma	PPARG	A2, A4, EC5, EC17, MF1, MF2, MF3, MF4, MF16, MF17, MF18MF22
Protein kinase C alpha type	PRKCA	A2, A3
Protein kinase C beta type	PRKCB	A2, MF4, MF13
Protein kinase C delta	PRKCD	MF13
Protein kinase C epsilon	PRKCE	MF13
Protein kinase C theta	PRKCQ	MF13
Phosphatidylinositol-3,4,5-trisphosphate 3-phosphatase and dual-specificity protein phosphatase PTEN	PTEN	A2, MF4
Prostaglandin G/H synthase 1	PTGS1	A2, A3, A4, EC2, EC3, EC4, EC7, EC9, EC10, EC18, MF1, MF2, MF3, MF4, MF6, MF7, MF12, MF22, MF23, MF24
Prostaglandin G/H synthase 2	PTGS2	A1, A2, A3, A4, EC2, EC3, EC4, EC5, EC7, EC9, EC10, EC11, EC12, EC14, EC15, EC16, EC17, EC19, EC22, EC24, MF1, MF2, MF3, MF4, MF10, MF12, MF17, MF18, MF21, MF22, MF23, MF24, MF25
mRNA of Protein-tyrosine phosphatase, non-receptor type 1	PTPN1	EC13, MF3, MF10, MF11, MF13, MF25
RAF proto-oncogene serine/threonine-protein kinase	RAF1	A2, MF3
Retinoic acid receptor alpha	RARA	MF3
Retinoic acid receptor beta	RARB	MF3
Retinoblastoma-associated protein	RB1	A2
Transcription factor p65	RELA	A2, A4, MF4
Rho-associated protein kinase 1	ROCK1	MF3
Nuclear receptor ROR-gamma	RORC	MF3
Runt-related transcription factor 2	RUNX2	A2
Retinoic acid receptor RXR-alpha	RXRA	A2, EC3, EC9, EC15, MF1, MF3, MF4, MF6
Sodium channel protein type 5 subunit alpha	SCN5A	A2, A3, EC2, EC3, EC5, EC7, EC9, EC10, EC16, EC19, EC24, MF1, MF2, MF6, MF21, MF22
E-selectin	SELE	A2, A4

P-selectin	SELP	MF4
Plasminogen activator inhibitor 1	SERPINE1	A2
Solute carrier family 2, facilitated glucose transporter member 4	SLC2A4	A2, A4
Sodium-dependent serotonin transporter	SLC6A4	A3, EC2, EC3
Antileukoproteinase	SLPI	A4
Acyl coenzyme A:cholesterol acyltransferase 1	SOAT1	EC13, EC18
Superoxide dismutase [Cu-Zn]	SOD1	A2
Osteopontin	SPP1	A2
Tyrosine-protein kinase SRC	SRC	MF4
Matriptase	ST14	MF11
Signal transducer and activator of transcription 1-alpha	STAT1	A2, A4
Tyrosine-protein kinase SYK	SYK	EC1
Telomerase reverse transcriptase	TERT	MF3
Transforming growth factor beta-1	TGFB1	A2, A3
TGF-beta receptor type I	TGFBR1	EC1
Thrombomodulin	THBD	A2
Toll-like receptor (TLR7)	TLR7	EC1
Tumor necrosis factor	TNF	A2, A4, MF13
Tumor necrosis factor receptor superfamily member 1A	TNFRSF1A	MF4
Tumor necrosis factor receptor superfamily member 1B	TNFRSF1B	MF4
DNA topoisomerase 2-alpha	TOP2A	A2
Cellular tumor antigen p53	TP53	A2
Tyrosinase	TYR	EC18
Vascular cell adhesion protein 1	VCAM1	A2, A4
Vascular endothelial growth factor A	VEGFA	A1, A2
Xanthine dehydrogenase/oxidase	XDH	A2, A4

Table S3. The information of active ingredients including 2D structure.

ID	Name	2D Structure	Database
A1	beta-carotene	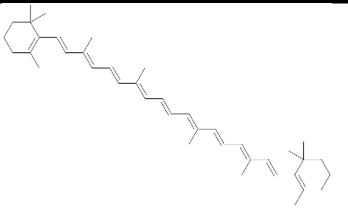	TCMSP
A2	quercetin	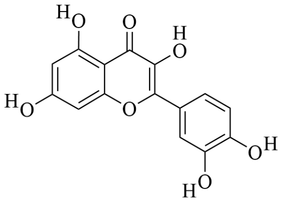	TCMSP
A3	beta-sitosterol	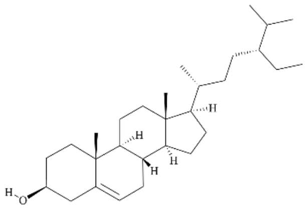	TCMSP
A4	kaempferol	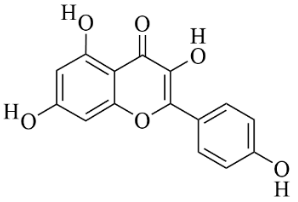	TCMSP
EC1	Dehydrodieugenol	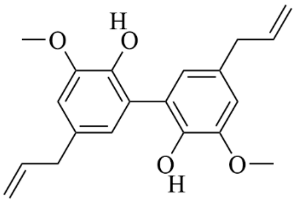	TCMSP
EC2	(A1-)-Tabernemontanine	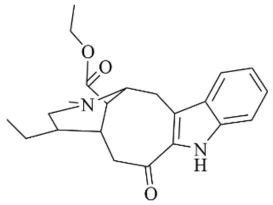	TCMSP
EC3	(9R)- 6'-methoxycinchonan-9-ol	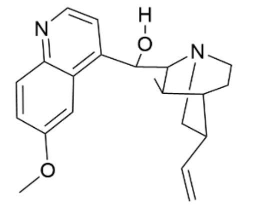	TCMSP

MF1	Tetramethoxyluteolin	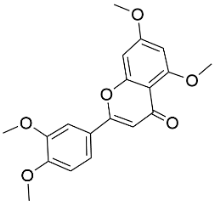	TCMSP
MF2	Iristectorigenin A	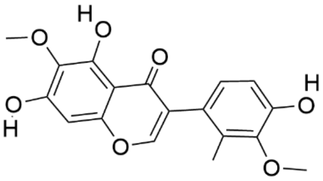	TCMSP
MF3	icosa-11,14,17-trienoic acid methyl ester	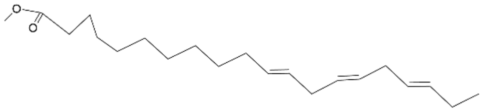	TCMSP
MF4	arachidonic acid	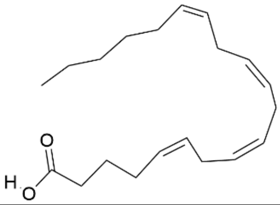	TCMSP

Table S4. The softwares and databases used in this study.

Name	Version	The url
TCMSP		https://www.tcmsp-e.com/
Pubchem		https://pubchem.ncbi.nlm.nih.gov/
SwissTargetPrediction		http://swisstargetprediction.ch/
GeneCards		https://www.genecards.org/
Cytoscape	3.8.1	
UniProt		https://www.uniprot.org/
Venny	2.1	https://bioinfogp.cnb.csic.es/tools/venny/
String	11.5	https://www.string-db.org/cgi/input?sessionId=bSwSO9xSIj50&input_page_active_form=multiple_identifiers
Metascape		https://metascape.org/gp/index.html#/main/step1
Chem 3D	19.0	
RCSB PDB		https://www.rcsb.org/
Sybyl-X	2.1.1	

Table S5. KEGG pathway analysis of MFEC to treat immune suppression.

Term ID	Description	P Value	Enrichment	Count
hsa05200	Pathways in cancer	5.22711E-37	19.63257421	82
ko04933	AGE-RAGE signaling pathway in diabetic complications	5.89259E-24	51.5409487	37
ko05418	Fluid shear stress and atherosclerosis	2.43812E-23	38.84700359	40
hsa04933	AGE-RAGE signaling pathway in diabetic complications	2.68872E-23	47.68741983	37
hsa05418	Fluid shear stress and atherosclerosis	5.55178E-23	37.27212507	40
hsa05161	Hepatitis B	5.89127E-23	32.72366235	42
hsa05215	Prostate cancer	7.5885E-23	48.67301038	36
hsa05167	kaposi sarcoma-associated herpesvirus infection	1.04074E-20	28.43440469	40
ko05215	Prostate cancer	1.14496E-20	50.72436331	32
hsa05166	Human T-cell leukemia virus 1 infection	1.53341E-20	21.03664008	45
hsa04151	PI3K-Akt signaling pathway	3.87349E-20	17.41981424	48
hsa05163	human cytomegalovirus infection	1.17483E-19	23.55908905	41
hsa05160	Hepatitis C	1.21346E-19	30.19262675	37
ko04151	PI3K-Akt signaling pathway	1.26524E-18	17.74240339	44
ko05166	HTLV-I infection	1.78412E-18	21.5479473	40
hsa04668	TNF signaling pathway	6.65578E-18	37.17489344	31
hsa05205	Proteoglycans in cancer	9.97559E-18	23.40621065	37
hsa05212	Pancreatic cancer	1.08881E-17	47.09014825	28
ko05205	Proteoglycans in cancer	1.40921E-17	24.45638945	36
ko04668	TNF signaling pathway	1.53644E-17	38.30746187	30
